# Case Report: Gastric glomus tumor with long-term dynamic multimodal imaging findings

**DOI:** 10.3389/fonc.2026.1932357

**Published:** 2026-09-03

**Authors:** Yujun Jiang, Wenli Lian, Xiaochen Dong, Xueyan Jia, Qingxia Xu, Min Wang, Zhongcheng Hang, Xuan Yang

**Affiliations:** 1 College of Medical Imaging and Laboratory, Jining Medical University, Jining, Shandong, China; 2 Department of Radiology, Jining No.1 People’s Hospital Affiliated to Shandong First Medical University, Jining, Shandong, China; 3 Department of Pathology, Jining No.1 People’s Hospital Affiliated to Shandong First Medical University, Jining, Shandong, China; 4 Department of Spinal Surgery, Jining No.1 People’s Hospital Affiliated to Shandong First Medical University, Jining, Shandong, China

**Keywords:** case report, computed tomography, endoscopic ultrasonography, gastric glomus tumor, magnetic resonance imaging

## Abstract

Gastric glomus tumor (GGT) is a rare mesenchymal neoplasm with nonspecific clinical and imaging features, often leading to preoperative misdiagnosis. Its longitudinal imaging evolution remains poorly characterized. Herein, we report a 51-year-old man with a GGT in the gastric antrum who underwent 6-year multimodal imaging follow-up, including CT, MRI, and serial endoscopic ultrasonography (EUS). The lesion presented as a well-circumscribed subepithelial nodule with marked and persistent enhancement on CT. It showed slightly low signal intensity on T1-weighted imaging, slightly high signal intensity on T2-weighted imaging, and mild early arterial enhancement followed by persistent marked enhancement on dynamic contrast-enhanced MRI. Over the 6-year follow-up, the lesion showed an overall increase in size and a transition from a homogeneous to a heterogeneous hypoechoic appearance on serial EUS. It was initially misdiagnosed as a gastrointestinal stromal tumor. Histopathology confirmed the diagnosis of GGT. This case highlights that GGT may exhibit characteristic yet dynamic imaging features on long-term follow-up. Recognition of these features across modalities may improve preoperative diagnostic accuracy.

## Introduction

Glomus tumor is a mesenchymal neoplasm arising from modified smooth muscle cells of the glomus body, typically located in the subcutaneous soft tissues of the distal extremities (1). Gastric glomus tumor (GGT) is an extremely rare entity that is predominantly benign, with malignancy reported in 1% of cases (2). Clinically, GGT lacks specific manifestations, with epigastric pain being the most common symptom (3). Due to its rarity, knowledge of the imaging characteristics of GGT is largely based on isolated case reports (3–5) and remains limited. Although the number of reported cases has increased in recent years (6, 7), most studies are cross-sectional and rely on single imaging modalities, providing limited understanding of its longitudinal imaging evolution. Imaging features of GGT reported by previous studies are nonspecific and overlap with gastrointestinal stromal tumor (GIST), neuroendocrine tumor (NET), heterotopic pancreas, and other gastric subepithelial lesions, leading to frequent preoperative misdiagnosis. To address this gap, we report a GGT in a patient with a 6-year longitudinal multimodal imaging follow-up, including CT, MRI, and serial endoscopic ultrasonography (EUS), providing a rare opportunity to illustrate its temporal imaging evolution across different modalities.

## Case presentation

**Figure 1:**
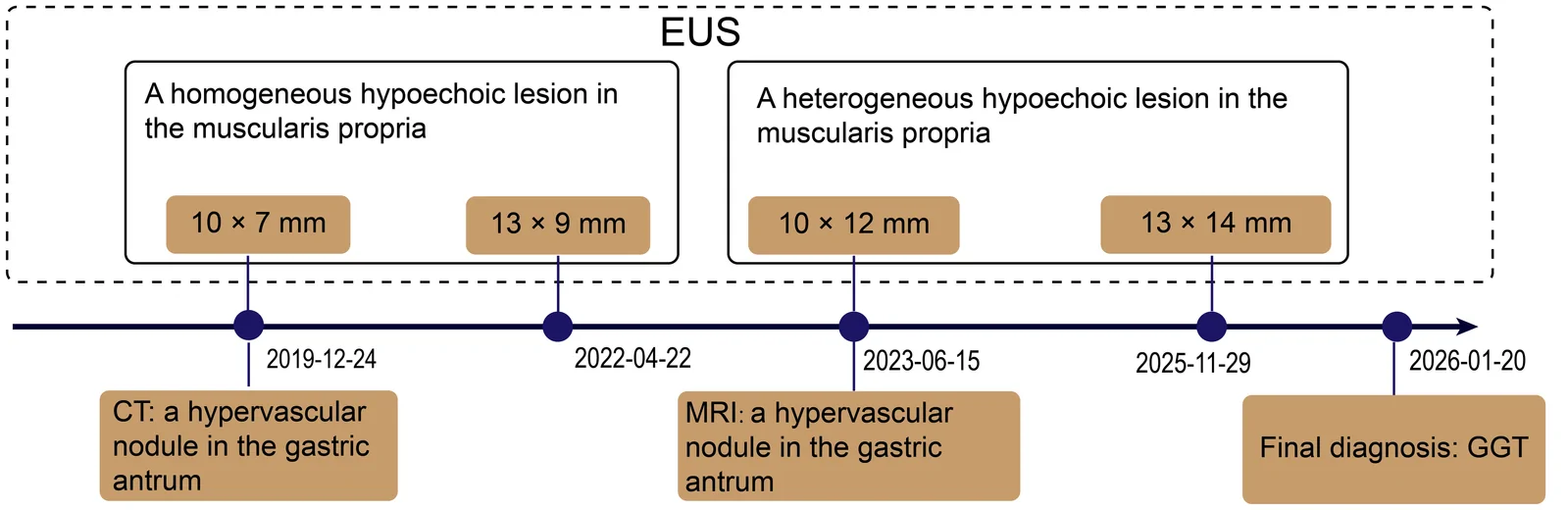
Timeline of imaging examinations. CT and endoscopic ultrasonography (EUS) were performed at baseline, followed by MRI and serial EUS over the 6-year follow-up. The lesion showed an overall increase in size and changed from a homogeneous to a heterogeneous hypoechoic appearance on serial EUS. It was preoperatively diagnosed as a gastrointestinal stromal tumor, and the final diagnosis of gastric glomus tumor (GGT) was established by postoperative histopathology.

A 51-year-old man presented to our hospital in December 2019 with a 2-month history of upper abdominal discomfort. The patient subsequently underwent unenhanced and contrast-enhanced abdominal CT together with EUS, followed by multiple imaging examinations over the next 6 years. A detailed timeline of imaging examinations is provided in **Figure 1**.

**Figure 2:**
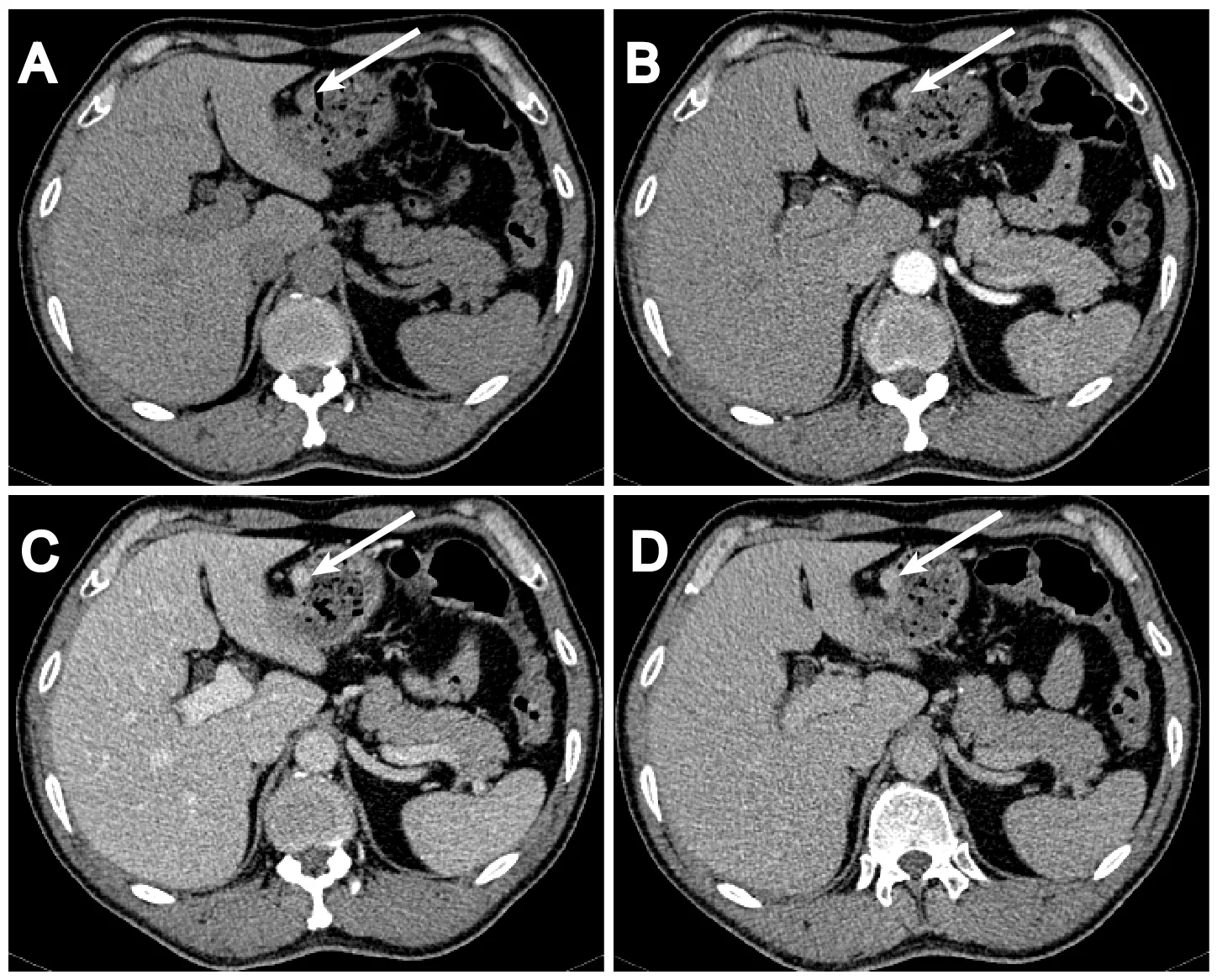
Unenhanced and contrast-enhanced CT findings of the lesion. **(A)** Unenhanced CT shows a well-circumscribed nodule (11 × 9 mm) in the anterior wall of the gastric antrum. **(B–D)** Contrast-enhanced CT demonstrates marked enhancement in the arterial phase with persistent enhancement in the portal venous and delayed phases. The lesion is indicated by the white arrow.

The initial CT demonstrated a well-circumscribed subepithelial lesion in the anterior wall of the gastric antrum (11 × 9 mm), appearing as a homogeneous soft-tissue nodule. It measured 49 HU on unenhanced images and showed marked arterial enhancement (82 HU) with persistent enhancement in the portal venous (118 HU) and delayed phases (97 HU) (**Figure 2**). EUS performed at the same time demonstrated a homogeneous hypoechoic lesion arising from the muscularis propria in the gastric antrum, with an intact overlying mucosa, confirming a deep-layer origin.

The lesion was subsequently followed with serial EUS examinations in April 2022, June 2023, and November 2025. In addition, unenhanced and contrast-enhanced MRI was performed in June 2023. Over the 6-year follow-up, the lesion showed an overall increase in size.

**Figure 3:**
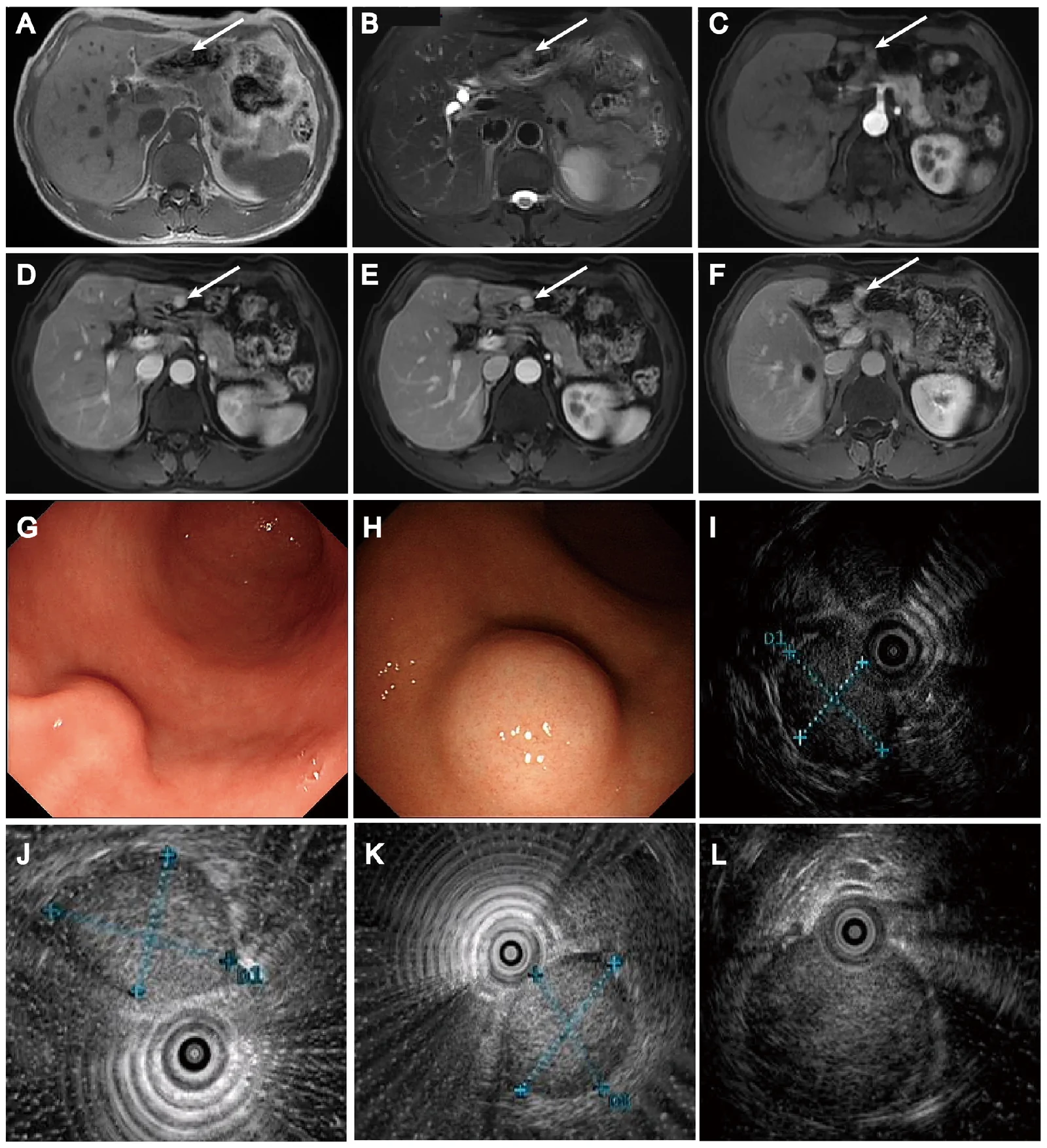
MRI and EUS findings of the lesion. **(A)** Axial T1WI demonstrates a slightly hypointense lesion in the anterior wall of the gastric antrum. **(B)** Axial T2WI shows a slightly hyperintense lesion. **(C, D)** Dynamic contrast-enhanced MRI demonstrates mild enhancement of the lesion in the early arterial phase and progressively increased enhancement in the late arterial phase. **(E, F)** The lesion shows persistent enhancement in the portal venous and delayed phases. The lesion is indicated by the white arrow. **(G, H)** Conventional endoscopic examinations performed in 2019 and 2025 demonstrate a persistent subepithelial lesion in the gastric antrum. **(I–L)** Serial EUS images obtained from 2019 to 2025 show a lesion arising from the muscularis propria. Over this period, the lesion showed an overall increase in size and changed from a homogeneous hypoechoic appearance to a heterogeneous hypoechoic appearance.

MRI in June 2023 showed a round nodule with slightly low signal intensity on T1-weighted imaging (T1WI) and slightly high signal intensity on T2-weighted imaging (T2WI). On dynamic contrast-enhanced imaging, the lesion demonstrated mild enhancement in the early arterial phase and marked enhancement in the late arterial phase, followed by persistent enhancement in the portal venous and delayed phases (**Figure 3**).

Over time, the lesion showed a gradual transition from a homogeneous hypoechoic appearance to a heterogeneous hypoechoic appearance on serial EUS (**Figure 3**). Given the overall imaging findings, the lesion was preoperatively diagnosed as GIST, and surgical resection was performed.

**Figure 4:**
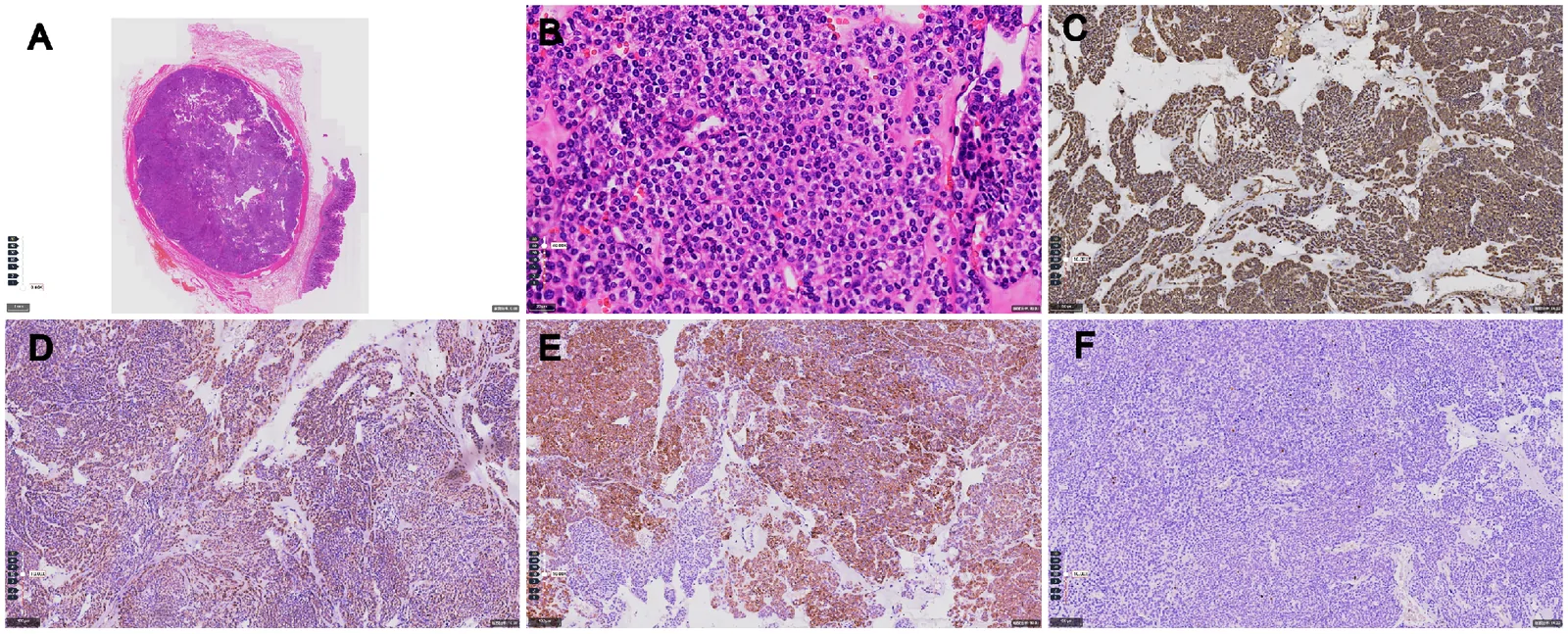
Gross pathological, histological, and immunohistochemical findings. **(A)** Gross specimen shows a well-circumscribed 1.4-cm nodule originating from the muscularis propria. **(B)** Hematoxylin and eosin staining (×20) demonstrates uniform round tumor cells arranged around vascular channels. **(C–E)** Immunohistochemistry shows positivity for smooth muscle actin (SMA), calponin, and synaptophysin (Syn). **(F)** Ki-67 labeling index is low (approximately 1%). These findings confirm the diagnosis of GGT.

Gross pathological examination confirmed that the lesion was located within the muscularis propria, forming a 1.4 cm well-circumscribed nodule. Histologically, tumor cells were arranged around vascular channels. Immunohistochemical staining showed positivity for synaptophysin (Syn), smooth muscle actin (SMA), caldesmon, calponin, and vimentin, whereas chromogranin A (CgA), CD56, cytokeratin pan (CKpan), and cytokeratin 19 (CK19) were negative. CD34 was positive in vessels, and the Ki-67 labeling index was approximately 1% (**Figure 4**). Taken together, the final diagnosis was GGT.

## Discussion

GGT is a rare mesenchymal tumor that is frequently misdiagnosed preoperatively due to overlapping imaging features with other gastric subepithelial lesions. Based on a review of the literature and our case, lesion location, morphology, enhancement pattern, and MRI signal characteristics may aid in differentiating GGT from other subepithelial lesions. GGTs typically occur in the gastric antrum and tend to present as round or ovoid lesions with a relatively low long-to-short axis ratio (7, 8), which is consistent with our case. A key imaging feature of GGT reported in the literature, based on CT imaging, is persistent marked enhancement (3, 7). Consistent with previous reports, contrast-enhanced CT in our case demonstrated marked arterial enhancement with persistence into the delayed phase, reflecting the rich vascularity of the tumor.

To our knowledge, this is the third report describing the imaging features of benign GGT on MRI (6, 9). All three studies consistently reported that GGTs exhibit slightly low signal intensity on T1WI and slightly high signal intensity on T2WI, which may serve as a supportive imaging feature. On dynamic contrast-enhanced imaging, the lesion showed mild early arterial enhancement with marked late arterial enhancement, followed by persistent enhancement in the portal venous and delayed phases, thereby providing a more detailed depiction of the dynamic enhancement pattern than previously reported.

Previous studies have generally described GGTs as hypoechoic lesions on EUS (8, 10); however, a small proportion of cases have reported iso- or hyperechoic appearances (11). In addition, both homogeneous and heterogeneous echotextures have been documented in the literature (7, 10). Our longitudinal follow-up provides additional insight into the temporal evolution of these features within the same lesion, demonstrating a transition from a homogeneous hypoechoic appearance to a heterogeneous hypoechoic appearance during the 6-year follow-up. This finding suggests that differences in echotexture may, at least in some cases, reflect temporal evolution of the lesion rather than only intertumoral variability.

The longitudinal findings may also have practical implications for clinical assessment and surveillance. The transition from a homogeneous to a heterogeneous hypoechoic appearance on EUS suggests that this change alone does not necessarily indicate aggressive behavior (7). The slow overall growth observed over 6 years is consistent with a previous report of gradual enlargement of a benign GGT during follow-up (10). In another study, benign GGTs showed annual growth rates ranging from 0% to 7.6% (median, 1.3%), which the authors regarded as essentially stable, whereas malignant GGTs demonstrated markedly higher annual growth rates of 22.5%–35.7% (median, 27.1%) (6). Taken together, evidence from our case and previous reports suggests that benign GGTs may also grow, but generally at a slower rate, and that growth rate may be more informative than the presence or absence of growth in differentiating benign from malignant GGTs. Furthermore, recognition of the slow growth pattern together with characteristic multimodal imaging features may facilitate the differentiation of GGTs from GISTs during longitudinal follow-up, as a subset of GISTs may demonstrate measurable growth over time (12). Nevertheless, further longitudinal studies are needed to establish recommendations for conservative management of GGT.

The differential diagnosis of GGT includes GIST, NET, heterotopic pancreas, gastric schwannoma, leiomyoma, hemangioma or vascular malformation, paraganglioma, and hypervascular metastasis. GISTs more commonly occur in the gastric fundus and body and typically demonstrate moderate and persistent enhancement, with low signal intensity on T1WI and high signal intensity on T2WI (7, 13). Accurate differentiation of GGT from GIST, a much more common gastric mesenchymal tumor, is clinically important because a diagnosis of GIST may necessitate a different preoperative assessment and surgical strategy (14–16). NETs typically appear as multiple well-circumscribed round nodules located in the gastric fundus and body, demonstrating marked arterial phase hyperenhancement (17). Heterotopic pancreas most frequently arises in the gastric antrum and often presents as an elongated lesion with a long-to-short axis ratio >1.4. On unenhanced MRI, it characteristically shows signal intensity similar to that of the orthotopic pancreas, exhibiting intrinsically high signal intensity on pre-contrast T1WI (18). A central umbilication may also be observed as a characteristic morphologic feature (3). Gastric schwannomas commonly occur in the gastric body and typically appear as well-circumscribed, homogeneous, low-attenuation masses on unenhanced CT, with gradual and relatively uniform enhancement on contrast-enhanced CT (3). On MRI, they usually show low-to-intermediate signal intensity on T1WI, high signal intensity on T2WI (19). Gastric leiomyomas often arise in the gastric cardia and typically exhibit an endophytic growth pattern, with relatively mild and homogeneous progressive enhancement (20). Gastric hemangiomas may demonstrate marked vascular enhancement on contrast-enhanced CT, and the presence of phleboliths may provide an additional diagnostic clue (21). Gastric paragangliomas are exceedingly rare. To our knowledge, only two reported cases have included imaging findings, describing either focal thickening of the gastric antral wall (22) or a cystic lesion in the gastric antrum (23). In functional tumors, catecholamine-related symptoms, including episodic hypertension, palpitations, headache, or diaphoresis, may provide an additional diagnostic clue. Hypervascular gastric metastases, particularly those from renal cell carcinoma, may present as intraluminal polypoid masses with marked enhancement on CT, and early enhancement followed by washout may provide an additional imaging clue (24).

## Conclusion

To our knowledge, this is the first report describing long-term, dynamic, multimodal imaging follow-up of GGT. A combination of imaging features, including a round lesion in the gastric antrum, a characteristic dynamic enhancement pattern, slightly low signal on T1WI, slightly high signal on T2WI, and a transition from a homogeneous to a heterogeneous hypoechoic appearance on serial EUS, may support the diagnosis of GGT. Recognition of these features may improve preoperative diagnostic accuracy and reduce misclassification.

## Data Availability

The original contributions presented in the study are included in the article/supplementary material. Further inquiries can be directed to the corresponding authors.
